# La Salle County Medical Society

**Published:** 1854-03

**Authors:** J. O. Harris

**Affiliations:** Secy.; Ottawa


For the N. W. Med. & Surg. Journal.

*La Salle County Medical Society.*

A regular meeting of this Society was held at Ottawa, on the 1st inst.

At that time Drs. J. Hard and J. Higgins were appointed delegates to the American Medical Association; Drs. J. Stout and T. Hay, substitutes ; and Drs. J. O. Harris and T. Hay delegates to the State Medical Society.

Drs A. Hard and R. McArthur having presented their credentials, were admitted as members of the Society, and Drs. W. A. Sanger, L. Eastman, —— Wooley and H. H. Hinman, were admitted as honorary members. A vote was passed authorising the admission of Dr. A. H. Howland, whenever he should present himself with the proper credentials.

Dr. A. Hard read an interesting paper on Diagnosis, and D. L. Kirwin exhibited his improved fracture box, and explained his manner of using the same.

Dr. Harris introduced the following resolution, viz.: Resolved that the members of this society counsel only with each other, or with physicians of good standing in the profession who have not had an opportunity of becoming members. After some discussion the resolution was laid on the table until the next meeting.

The utmost harmony and good feeling prevailed, and the meeting passed off very pleasantly indeed to all who were present.

Various matters of professional interest were talked of and discussed, after which the Society adjourned to meet at Peru, on the first Monday in June next.

J. O. HARRIS, Secy.

OTTAWA, March 3, 1854.

